# To the Right Honourable the Lords Commissioners of His Majesty's Treasury

**Published:** 1810-10

**Authors:** 


### To the Editors of the Medical and Physical Journal.

GENTLEMEN,

IF you deem the accompanying memorial worthy of being inserted in the Medical and Physical Journal, I shall be glad to see it published, and think it of sufficient importance to be submitted to the attention of your numerous readers. A copy of the Memorial, together with a sketch of the Bill for amending Medical Education and Practice, and a message from the Treasury, have, I am told, been forwarded to all the Medical Corporate Bodies in England, Scotland, and Ireland. In this message, the Colleges are desired to take the subject under consideration, and report their sentiments to the Lords of the Treasury. My information may be relied on. It was derived immediately from the Fellow of a Royal College, who had seen the documents. Of course none of the Public Bodies will presume to pass over the application in silence, or omit to declare their sentiments, after receiving an application from such authority. I am now convinced that something will be attempted next winter to ameliorate the condition of the faculty, and make them better entitled to the confidence of their employers. I have been repeatedly told that the Members who are to propose and second the motion for receiving the Bill in the House of Commons are already appointed.—The subject is of such vital interest, both to the profession and society at large, that I am desirous to see it obtain its due consideration. It is under this impression that I have solicited a place in your Journal for this Letter, and Dr. Harrison's Memorial.

I am, Gentlemen,

YOUR CONSTANT READER.

### *To the Right Honourable the Lords Commissioners of his Majesty's Treasury.*

The Memorial and Representation of EDWARD HARRISON, M. M. D. F. A. S. Ed. &c.

THE representation which your memoralist humbly presumes to offer to the consideration of your Lordships, having for its object the application of a practical remedy to the numerous abuses which have long and confessedly existed in the various branches of the medical profession, it is scarcely necessary to add, that the subject involves the vital interests of the whole community, whether collectively or individually considered dered, and forms an object of the most interesting contemplation, both to the legislator and the political economist.

That an individual should now offer a system of reform in so important a branch of jurisprudence, may require explanation. He presents himself to your attention, as the representative of a respectable body of medical gentlemen, who, during the last six years, have devoted much time and attention to the obtaining of that information which is necessary in such an undertaking; and he presumes to hope that the materials thus collected, and the consideration already bestowed on this important object, as well by themselves, as by the high legal authorities which they have consulted, may form the basis of a superstructure highly beneficial to the present and future generations.

Though conscious of the magnitude, he is not insensible to the difficulties of the attempt, and probably the most prominent of those difficulties is, that of regulating by any theoretic ideas, practices which, whether regular or irregular, have in point of fact the sanction of ages for their continuance: the first sensation generally felt on such occasions, is a repugnance to innovation.

If, however, this sentiment had universally prevailed, the community would not at this day have experienced the practical utility so universally admitted to result from those parliamentary restrictions which have already regulated the practice in other professions. It may indeed furnish matter of surprise, that while the wisdom of the legislature has been successfully applied to the protection of the property of the subject, a science so materially affecting the life of each individual, should have received so little of its attention, as never to have been an object of its consideration since the time of Henry the 8th. No man can presume to tender his services for the recovery or protection of his neighbour's property, in a court of law, without offering at least a species of security for his abilities, in the provision which the law has made for his education and admission to that profession; but in the more important concerns of health and life, no such security is afforded to the employer; he has no possible access to know under what authority the numerous pretenders to medicine make him a tender of their services; hence a profession, honourable and useful in itself, is disgraced by needy and ignorant adventurers. To such an extent has the mischief prevailed, that in a considerable district to which particular inquiry has been directed, it is ascertained, that not more than about one in nine of those who publicly practise for gain, has passed through any regular course of education to qualify qualify him for the duties; and there is good reason to believe from repeated inquiries, by means of circular applications, that the state of medical practice is equally defective in other parts of the British dominions.

Your memoralist can assert, without the fear of contradiction, that there is no corporate or other body in this kingdom short of the legislature itself capable of applying an adequate, or indeed any remedy to this great and increasing evil.

It would be inconvenient, in an address of this nature, to attempt a particular detail of the mischiefs resulting from the present state of medical practice; the public benefits to be expected from a temperate reform; the regulations by which it is hoped that such benefits might be secured, or the steps already taken with a view to the accomplishment of this great object; but, if your memoralist may be permitted to refer to his printed "Address to the Lincolnshire benevolent Medical Society," lately published, he humbly hopes that the attention of his Majesty's Government, thus drawn to the heads* of a bill, which he has the honour of submitting to their consideration, may facilitate such an enactment in parliament as would prove highly beneficial to the state.

The bill merely aims at general regulation; and so far from seeking to infringe on the rights of any of the learned chartered bodies in his Majesty's dominions, its tendency is to give additional weight and importance to those establishments whose consequence is identified with that of the great body of medical practitioners.

Its objects are not complex, nor do they present any particular difficulties in the execution; it seeks not to place the present race of practitioners, under any odious restraint which might operate as an harsh and *ex post facto* law to many of them.

Its prominent and leading features are,

1st, To insure to the public the positive fact that any one who may hereafter offer himself to their employment under any of the denominations applied to the practice of physic or surgery, shall have devoted a reasonable time to his education in that department in which he shall profess to practise or gain.

2dly, To secure a faithful and national register of the accre-

* The conductors of the Bill do not undertake more in the first act than to lay the foundation of reform. In fact, the subject has been so little considered by the faculty themselves, and each is so desirous to promote his own views rather than the general good of his profession and of the community, that until a large proportion can be brought to consider the matter dispassionately, it will be dangerous and highly imprudent to enter upon minutiæ.

dited dited practitioners in the different branches of those professions.

3dly, To establish a school of medicine in this kingdom, and to improve other schools on a rational and practicable basis, out of funds to be provided by the body itself.

Your memoralist is fully impressed with the belief that a bill, founded on some such basis, and under such modifications as to the wisdom of Parliament may seem expedient, would, if passed into a law, tend greatly to the reduction of human misery, the preservation of many valuable lives, and the consequent advantage and happiness of the whole community.

But, as investigation and inquiry on such a subject are peculiarly desirable, your memoralist humbly hopes, that should this his Representation be deemed worthy of your Lordships' attention, you will be pleased to direct a copy of the accompanying sketch of a bill to be sent, under the high sanction of your names, to each of the Medical Corporate Bodies* in the United Kingdom, whose titles are under-written, and to request that answers be returned as soon as may be convenient, that the sentiments of those respectable bodies may be fully understood and considered, as applying to the necessity of Medical Reform in general, and to the provisions of the proposed Bill in particular.

(Signed) E. HARRISON.

---

### *To the Editors of the Medical and Physical Journal.*

GENTLEMEN,

YOUR candour will, I doubt not, permit me to correct a great error into which Mr Royston has fallen, I dare say from inadvertence, in his account of my "Reports on the Effects of Regimen in Cancer," published in your Journal for July last.

---

* LIST OF THE MEDICAL PUBLIC BODIES.

1. The Royal College of Physicians of London.
2. The Royal College of Surgeons of London.
3. The Company of Apothecaries of London.
4. The Royal College of Physicians of Dublin.
5. The Royal College of Surgeons of Dublin.
6. The Company of Apothecaries of Dublin.
7. The Royal College of Physicians of Edinburgh.
8. The Royal College of Surgeous of Edinburgh.
9. The Faculty of Physicians and Surgeons of Glasgow.

